# The First Telomere-to-Telomere Chromosome-Level Genome Assembly of *Stagonospora tainanensis* Causing Sugarcane Leaf Blight

**DOI:** 10.3390/jof8101088

**Published:** 2022-10-16

**Authors:** Fu Xu, Xiuxiu Li, Hui Ren, Rensen Zeng, Zhoutao Wang, Hongli Hu, Jiandong Bao, Youxiong Que

**Affiliations:** 1 Key Lab of Sugarcane Biology and Genetic Breeding, Ministry of Agriculture and Rural Affairs, Fuzhou 350002, China; 2 College of Plant Protection, Fujian Agriculture and Forestry University, Fuzhou 350002, China; 3 State Key Laboratory for Managing Biotic and Chemical Treats to the and Safety of Agro-Products, Institute of Plant Protection and Microbiology, Zhejiang Academy of Agricultural Sciences, Hangzhou 310021, China

**Keywords:** *Stagonospora tainanensis*, sugarcane leaf blight, pathogenicity, Nanopore sequencing, genome assembly

## Abstract

The sexual morph *Leptosphaeria taiwanensis* Yen and Chi and its asexual morph *Stagonospora tainanensis* W. H. Hsieh is an important necrotrophic fungal phytopathogen, which causes sugarcane leaf blight, resulting in loss of cane tonnage and sucrose in susceptible sugarcane varieties. Decoding the genome and understanding of the basis of virulence is vitally important for devising effective disease control strategies. Here, we present a 38.25-Mb high-quality genome assembly of *S. tainanensis* strain StFZ01, denovo assembled with 10.19 Gb Nanopore sequencing long reads (~267×) and 3.82 Gb Illumina short reads (~100×). The genome assembly consists of 12 contigs with N50 of 2.86 Mb of which 5 belong to the telomere to telomere (T2T) chromosome. It contains 13.20% repeat sequences, 12,543 proteins, and 12,206 protein-coding genes with the BUSCO completeness 99.18% at fungi (*n* = 758) and 99.87% at ascomycota (*n* = 1706), indicating the high accuracy and completeness of our gene annotations. The virulence analysis in silico revealed the presence of 2379 PHIs, 599 CAZys, 248 membrane transport proteins, 191 cytochrome P450 enzymes, 609 putative secreted proteins, and 333 effectors in the StFZ01 genome. The genomic resources presented here will not only be helpful for development of specific molecular marker and diagnosis technique, population genetics, molecular taxonomy, and disease managements, it can also provide a significant precise genomic reference for investigating the ascomycetous genome, the necrotrophic lifestyle, and pathogenicity in the future.

## 1. Introduction

Sugarcane (*Saccharum* spp. hybrids), cultivated in more than 120 countries, is a crucial sugar crop accounting for 80% of the world’s and nearly 90% of China’s sugar production [1,2]. Similar to other crops, sugarcane is also exposed to many different diseases during cultivation. Among them, fungal diseases are the most serious due to the production of a large number of conidia, which can be transmitted by air, wind, and the splash of water during rain, and lead to the elimination of many elite cultivars [3,4,5,6]. Unlike the stalk-infected diseases, such as smut and pokkah boeng caused by *Sporisorium scitamineum* and *Fusarium* sp., respectively, which lead to serious economic losses almost every year in susceptible varieties, the foliar diseases are confined to leaves and outbreaks in susceptible varieties during monsoon, which increase wounds and humidity, a steady stream of wet weather and after the monsoon season. It means that the foliar diseases do not cause outbreaks every year and thus the economic repercussions caused by them are uncertain. Therefore, less attention has been paid to the foliar diseases especially for those with limited distribution, such as sugarcane leaf blight (SLB). However, we note an increasing concern of foliar diseases in sugarcane due to minor diseases becoming major diseases. For example, pokkah boeng was a minor disease in past decades [7], but it has become a major disease in India [8,9]. The first severe outbreaks of brown rust caused by *Puccinia melanocephala* on sugarcane were reported in 1978 in Florida [10] and recently reported in India [11]. Orange rust caused by *Puccinia kuehnii* was also considered a minor disease in most countries, including Australian before 1999; however, severe epidemics occurred in cultiver Q124 during 1999–2001, which resulted in 50% of yield losses, overall losses estimated to be A$150–210 millions [4], and an outbreak of this disease in America in 2007 [12]. Additionally, brown spot caused by *Cercospora longipes* was found to be alarming in India [13], peanut collar rot caused by *Aspergillus niger* in Asia [14,15], and brown stripe caused by *Helminthosporium stenospila* have been major diseases in China [16]. Similarly, SLB has been alarming recently in Yunnan and Guangxi provinces in China, due to the changed climate and the expansion of susceptible varieties, such as Guitang42, Taitang25, and Liucheng03-182 [16].

Sugarcane leaf blight caused by *Stagonospora tainanensis* was first reported in the year 1952 in Taiwan, China, and its asexual morph was first named *Cercospora tainanensis* [17] and then changed to *S. tainanensis* W. H. Hsieh, according to the further study on the isolates and inoculation, together with the pathogenic morphological features [18]. It occurred through the year in the east coast area with high rainfall, but not in the west coast area with less rain fall. The prevalence conditions for SLB were similar to that of the disease of Stagonospora nodorum blotch caused by *Stagonospora. nodorum* in wheat [19,20] of which the sexual morph is *Leptosphaeria nodorum* [21]. For a long time, research on SLB has been limited to pathogen isolation and identification, although it is one of the most harmful fungal diseases threatening the sugarcane industry and causing high cane yield and sugar losses in susceptible cultivars [17,22] because the pathogenic conidia are highly virulent, cause blight symptoms on sugarcane leaves, and result in loss of photosynthetic capacity [23]. Morphologically, *S. tainanensis* is an ascomycete, belonging to *Stagonospora* of Phaeosphaeriaceae within the class Dothideomycetes and the order Pleosporales. Its asci are slightly curved, 62–115 × 21–33 μm in size, born in scattered and dark brown perithecia [22]. Each ascus contains eight fusiform bent ascospores with one septum and its cylindrical conidia are straight to slightly curved in shape with three septa generally, containing three to eight oil droplets [20]. Recently, the efficient PCR detection technology of *S. tainanensis* and SLB were developed based on genomic information [24], and identification of SLB-resistance associated loci/genes were reported [25,26]. These will advance our understanding of the molecular mechanisms of pathogen infection and host resistance.

In the recent 10 years, the revolutionary progress of the third-generation sequencing (TGS) technologies led by PacBio and Oxford Nanopore Technology (ONT) has brought genome research into a new era [27,28]. Third-generation sequencing can produce long reads from 10 kb to 1 Mb, which dramatically reduce the time and cost for genome assembling and thus makes it possible to finish a high-quality non-model fungus genome assembly of approximately 50 Mb for a small lab [29]. For a model plant pathogenic fungi, such as *Pyricularia oryzae* and *Fusarium graminearum,* TGS-based chromosome-level reference genome and more than 100 genome assemblies are available in NCBI [30,31,32,33]. For fungi in the *Massarinaceae* family, only three species, including *Byssothecium circinans* [34], *Massarina eburnea* [35], and *Stagonospora* sp. [36], have been reported. However, in our studied genus *Stagonospora*, only *S. nodorum*, a model species of necrotrophic Pleosporales pathogens, had been sequenced. Additionally, *S. nodorum* genome was the first Dothideomycete genome, which was sequenced in 2005 and published in 2007 [37], and had a revolutionary impact on the understanding of this important pathogen and other fungal pathogens due to the limited genomic information available.

Until now, the genome of *S. tainanensis* has not been publicly available. In this study, we employed Nanopore sequencing and Illumina sequencing together to finish a near telomere-to-telomere chromosome-level genome assembly and RNA-seq based gene annotation of this necrotrophic infecting fungus *S. tainanensis* strain StFZ01. It can provide a more precise understanding of the pathogen and the fungal pathogenicity and can offer a series of putative proteins in the fungal pathogenesis, such as effectors, and it is thus beneficial for developing a new disease management strategy and for sugarcane improvement of leaf blight resistance.

## 2. Materials and Methods

### 2.1. Sample Preparation and Sequencing

**Figure 1:**
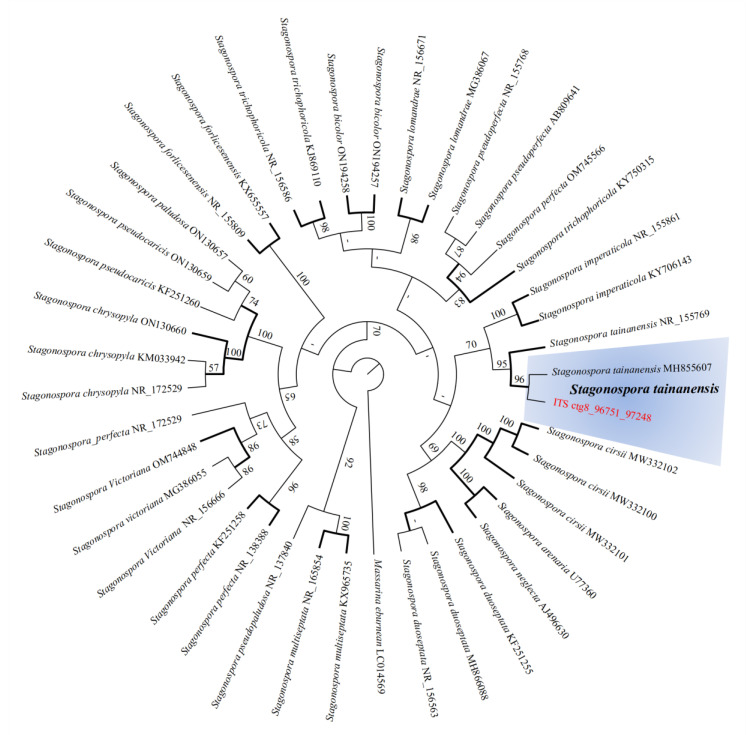
The species of strain StFZ01 was verified as *Stagonospora tainanensis* by maximum likelihood phylogenetic tree analysis (bootstrap = 1000) conducted by MEGA v11 (https://www.megasoftware.net/) (accessed on 17 August 2022) based on ITS sequences with high similarity collected from NCBI. Table S1 Summary of sequencing reads.

Sugarcane leaf blight-susceptible sugarcane cultivar Yuetang93-159 was planted in Fuzhou, China (26°5′0″ N, 119°13′45″ E) and the *S. tainanensis* strain StFZ01 was collected from its leaves with the typical symptoms of SLB. After the isolates were testified using ITS detection (Figure 1) and by pathogenic morphology [24], one assigned as StFZ01 was used for genome sequencing and analysis. The fresh mycelia cultivated on potato dextrose broth (PDB) media was collected for DNA and RNA extraction. For long-read genomic sequencing, high-quality genomic DNA was extracted using Ligation Sequencing Kit (SQK-LSK110), then BluePippin DNA size selection system was used to select large DNA fragments (>20 kb) for sequence library preparation following the manufacturer’s instructions, and the sequencing was conducted on *PromethION* sequencing platform from Oxford Nanopore Technologies (ONT). For Illumina short-read sequencing, genomic DNA and mRNA were extracted, purified, and prepared for sequencing libraries using Illumina DNA Prep Kits (Illumina, Inc., San Diego, CA, USA) and Illumina Stranded mRNA Prep (Illumina, Inc., San Diego, CA, USA), respectively. Illumina genomic DNA sequencing and RNA-seq were performed on the Illumina HiSeq 3000 sequencing platform (350 bp library and PE150 strategy).

### 2.2. Genome Size Estimation

The genome size of strain StFZ01 was estimated using GenomeScope v2.0 [38] based on the k-mer frequencies of genomic Illumina short reads (k = 21, p = 1) computed by KMC v3.1.1 [39].

### 2.3. De Novo Genome Assembly

The de novo assembly of ONT long reads was performed using NextDenovo v2.5.0 (https://github.com/Nextomics/NextDenovo) (accessed on 10 January 2022) with the “correct-then-assemble” strategy. Then, base errors (SNPs/Indels) of the draft genome assembly were fixed by NextPolish v1.4.0 [40] using both ONT long reads and Illumina short reads (task = best model) to generate a high-continuity and high-accuracy genome assembly of strain StFZ01.

### 2.4. Genome Completeness Assessment

The software Benchmarking Universal Single-Copy Orthologs (BUSCO) v5.3.2 [41] was used to evaluate the completeness of the genome assembly and annotated genes with the lineage dataset of fungi_odb10 (*n* = 758) and ascomycota_odb10 (*n* = 1706). Furthermore, the completeness of the genome assembly was also assessed by mapping of sequenced reads. RNA-Seq reads were aligned to the repeat-masked genome assembly using HISAT2 v2.2.1 [42]. The ONT long reads and genomic Illumina short reads were mapped to the unmasked genome assembly with minimap2 v2.21-r1071 [43] and BWA-MEM2 v2.2.1 [44], respectively (Table S1).

Telomeric repeats (5′-TTAGGG-3′)n or (5′-CCCTAA-3′)n were searched at both ends of the contigs. The gapless contig ending with telomeric repeats should be an telomere-to-telomere (T2T) chromosome [45].

### 2.5. Repeat Masking

Transposable element (TE) of the StFZ01 genome was annotated using a combination of ab initio and homology-based methods. First, a high-quality ab initio TE library was constructed with RepeatModeler v2.02 [46]. Next, RepeatMasker v4.1.2-p1 (http://repeatmasker.org/) (accessed on 20 April 2021) was applied to perform a homology-based TE search throughout the StFZ01 genome using the ab initio TE database. Finally, The StFZ01 genome was repeat-masked as the hard-masked (repeat sequences replaced with N) sequence used in mapping of RNA-seq reads and the soft-masking (repeat sequences masked as low case) genome for gene annotation.

### 2.6. Annotation of Protein-Coding Genes

Protein-coding genes were annotated using BRAKER v2.1.6 genome annotation pipeline [47], which integrates both ab initio gene predictions generated by Augustus v3.4.0 [48] and GeneMark-ET [49], and gene structure evidence, including fungal homologous protein sequences in fungi_odb10 (https://busco-data.ezlab.org/v5/data/lineages/) (accessed on 31 December 2021) as well as a de novo transcriptome assembly generated from RNA-seq data in this study.

### 2.7. Identification of Non-Coding RNAs

Transfer RNAs (tRNAs) were identified by tRNAscan-SE v2.0.9 with eukaryote parameters [50]. Ribosomal RNA (rRNA) and other non-coding RNAs (ncRNAs) were predicted using Infernal v1.1.4 [51] by searching against the RNA families database Rfam v14.8 [52].

### 2.8. Functional Annotation of Protein-Coding Genes

#### 2.8.1. General Functional Annotation

Gene Ontology (GO) and Pfam terms were grouped into categories based on results from InterProScan v5.55-88.0 [53]. EuKaryotic Orthologous Groups (KOG) were annotated by eggNOG-mapper v2 [54]. The predicted protein-encoding genes were assigned KEGG (Kyoto Encyclopedia of Genes and Genome) using KofamKOALA [55], then these KOs were used to reconstruct KEGG metabolic pathway maps at KEGG Mapper Reconstruction online web service (https://www.genome.jp/kegg-bin/find_pathway_object) (accessed on 8 June 2022).

#### 2.8.2. Fungal Pathogenicity-Related Gene Annotation

Annotation of pathogenicity-related genes was conducted by DIAMOND v2.0.11 [56] against a set of databases including PHI-base v4.12 (http://www.phi-base.org/) (accessed on 21 September 2021), dbCAN2 (https://bcb.unl.edu/dbCAN2) (accessed on 31 July 2022), and TCDBs (https://tcdb.org/) (accessed on 31 July 2022) for identification of pathogen–host interaction related genes (similarity ≥ 30% and alignment length ≥ 100 aa), carbohydrate-active enzymes (CAZys) (verified by HMMER v3.1b2 [57] via bCAN and dbCAN-sub databases, number of tools ≥ 2), and membrane transport proteins (similarity ≥ 50% and alignment length ≥ 200 aa), respectively.

The putative secreted proteins were identified following a pipeline in the previous study [30], of which proteins with signal peptide and without transmembrane helix were identified by SignalP v5.0 [58] and TMHMM v2.0 [59], respectively, and those with extracellular location were identified using ProtComp v9.0 from MolQuest v2.4 (Softberry Inc., New York, USA). Furthermore, these effectors were further scanned by EffectorP v3.0 [60] and divided into cytoplasmic and apoplastic effectors.

#### 2.8.3. Secondary Metabolite Biosynthetic Gene Clusters Analysis

Online web service (https://fungismash.secondarymetabolites.org) (accessed on 8 June 2022) of AntiSMASH v6.0 [61] for fungi was employed for identification of secondary metabolite biosynthetic gene clusters (SMBGCs).

### 2.9. Comparative Genomic Analysis

Whole-genome protein sequences of nine fungi species, including three of the *Massarinaceae* family and six of the *Pleosporaceae* familiy in the *Pleosporales* order, were downloaded from NCBI (Table S2). The longest proteins for each gene were selected and clustered using OrthoFinder v2.5.4 [51] with the following parameters: -S diamond, -M msa [62]. Single-copy core orthologous proteins were aligned using MAFFT v7.490 [63] and then the phylogenetic tree of species was constructed with FastTree v2.1.11 [64] and visualize by Interactive Tree Of Life (iTOL) v6.5.8 online services [65].

## 3. Results and Discussion

### 3.1. The Morphology of the Pathological Lesions and Pathogenic S. tainanensis Used for Genome Sequencing

**Figure 2:**
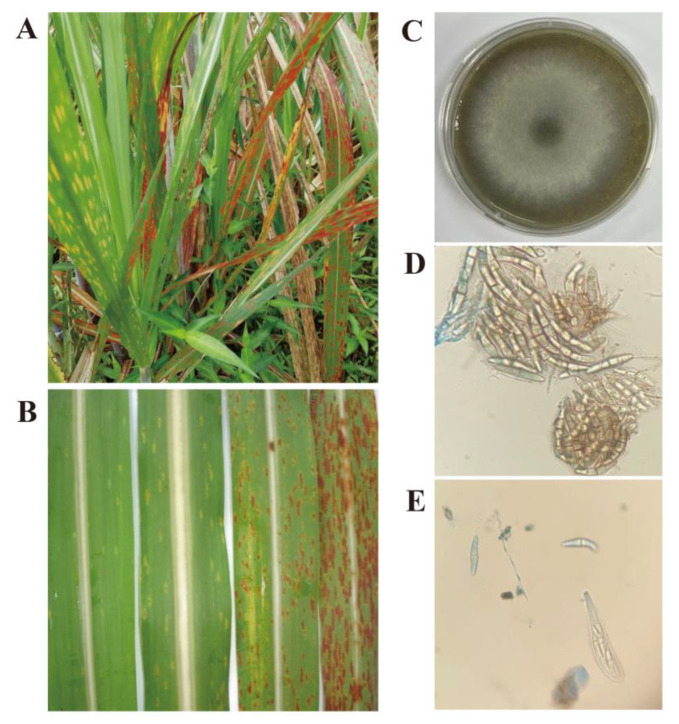
The phenotypic symptoms of sugarcane leaf blight (SLB) on sugarcane cultivar Yuetang93-159 initiated by *Stagonospora tainanensis* and the morphological characteristics of the strain StFZ01. (**A**) The phenotypic symptoms of SLB on plants. (**B**) Development of the lesions of SLB on leaves. (**C**) The pathogenic colony and mycelia. (**D**) The pathogenic conidia. (**E**) The sexual asci and ascospores.

The typical single mature or early-mature lesion of sugarcane leaf blight on the infected leaves caused by *S. tainanensis* is spindly and elongated, which is observed on variety Yuetang93-159 (Figure 2A). The color of pathological leaf tissues was found to change from early yellowish to yellow, then to red-brown (Figure 2B). Therefore, the typical symptoms of SLB are relatively easy to identify in later stages of lesion development (Figure 2B). The mycelia of the pathogenic *S. tainanensis* strain StFZ01 used for DNA isolating were collected from the culture growth on PDA agar medium (Figure 2C) and the pathogenic conidia (Figure 2D), and sexual asci and ascospores (Figure 2E) were observed.

### 3.2. Genome Sequencing and Assembly

**Figure 3:**
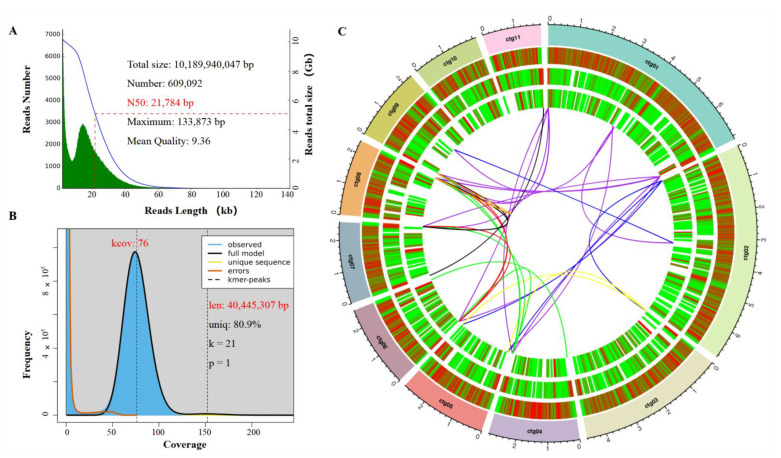
Genome features of *Stagonospora tainanensis* strain StFZ01. (**A**) Summary of ONT reads. (**B**) Genome size estimation with NGS genomic reads; (**C**) Circos plot of genome assembly features. Circles from outside to inside present contigs (1st circle, the smallest contig ctg12 was not shown), distribution of protein-coding genes (2nd), TEs (3rd), and putative secreted proteins (4rd) per 50 kb window size (color blue to red means number from low to high). The lines in the center of circle show the synteny blocks (≥10 kb) between different contigs.

After quality control, a total of 10.19 Gb clean ONT long reads (depth: ~267×, N_50_: 21,784 bp, maximum length: 133,873 bp) were used for de novo genome assembly, 3.82 Gb Illumina short reads (depth: ~100×, 2 × 150 bp) for estimation of genome size and polishing of draft genome assembly, and 6.08 Gb RNA-seq reads (2 × 150 bp) for gene annotation (Figure 3A and Table S1).

We estimated genome size of *S. tainanensis* strain StFZ01 to be 40,445,307 bp (Model fit = 98.13%, ploidy = haploid), based on k-mer distribution (k = 21, average k-mer depth 76×) of Illumina short reads (Figure 3B and Table S3). The estimated repeat is 19.09% (7,720,991 bp) of the genome sequence. The 38.25 Mb genome assembly (GC: 51.49%) of StFZ01 contained 12 contigs with N_50_ of 2.86 Mb (L_50_ = 4), N_90_ of 2.11 Mb (L_90_ = 10), and a maximum contig of 7.12 Mb (Table 1 and Figure 3C). The length of genome assembly is slightly smaller than the estimated genome size (94.58% of 40,445,307 bp). Its genome size is comparable to another species *S.* *nodorum* (37.21 Mb) in the same genus and *Bipolaris maydis* (36.23 Mb) causing Southern corn leaf blight [66], but smaller than the *Leptosphaeria maculans* (45.12 Mb), a pathogenic fungus closely related to *S. nodorum*, and much smaller than the genomes of *Colletotrichum higginsianum* (53.4 Mb) and *Colletotrichum graminicola* (57.4 Mb) [67].

A total of nine contigs (ctg1-ctg9) were found to start or end with telomeric repeat, (5′-TTAGGG-3′)n or (5′-CCCTAA-3′)n, of which five contigs (ctg1, ctg3, ctg5, ctg6, ctg7, and ctg9) contain telomeric repeats at both contig ends, indicating that these contigs reached perfect gapless T2T chromosome level [45] (Table S4).

### 3.3. Genome Quality Assessment

**Figure 4:**
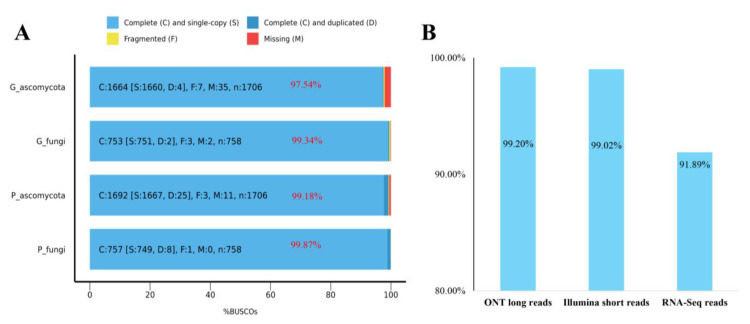
Genome completeness assessment of *Stagonospora tainanensis* strain StFZ01. (**A**) BUSCO completeness assessment of genome assembly and annotated proteins with the lineage dataset of fungi_odb10 and ascomycota_odb10. G means genome and P means proteins. (**B**) Genome completeness valued by mapping rate of different reads.

The BUSCO completeness values were estimated to be 99.34% at fungi (*n* = 758) and 97.54% at ascomycota (*n* = 1706) for genome assembly of StFZ01 (Figure 4A). All clean ONT long reads and Illumina genomic reads were aligned to unmasked genome assembly, and the mapping rate of ONT long reads and Illumina genomic reads are 99.20% and 99.02%, respectively (Figure 4B and Table S1). Furthermore, one RNA-seq sample of StFZ01 was mapped to repeat-masked genome assembly, and 91.89% (85.9% properly paired) of RNA-seq reads showed unique mapping to gene regions (Figure 4B and Table S1). All of these results attest to the high continuity and completeness of our assembled genome.

### 3.4. Repeat Analysis

Repetitive sequences were identified using a combination of ab initio and homology-based approaches. In total, 13.20% (5,048,126 bp) of the assembled StFZ01 sequences were annotated as repeat sequences (Table 1 and Table 2), which is less than the *C. graminicola* (22.3%) but more than the *C. higginsianum* (9.1%) [67]. Interspersed repeats, as the major component (90.41% of total repeats) were found to account for 11.28% of the genome, including 1,927,917 bp long terminal repeats (LTRs), 1,242,571 bp DNA transposons, 498,662 bp long interspersed nuclear elements (LINEs), 12,515 bp short interspersed nuclear elements (SINEs), and 1,242,571 bp unclassified interspersed repeats (Table 2). The dynamic polymorphism of repeat insertion in phytopathogenic fungi usually associated with virulence variations, hence, high frequent interspersed repeats will be candidate DNA markers for identification of different virulent strain, similar to Pot2 rep-PCR fingerprinting analysis in rice blast fungus [68]. In addition, we identified several types of non-coding RNAs, including 162 tRNAs, 142 rRNAs, and 48 other ncRNAs (Table 1).

### 3.5. Gene Structural Annotation

In total, 12,206 high-confidence protein-coding genes were predicted by the BRAKER2 pipeline, which was more than the *Magnaporthe grisea* PMg_Dl (10,218) though its genome size (38.25 Mb) was less than the *M. grisea* (47.89 Mb) [69], and this phenomenon was also observed in the other necrotrophic fungal pathogen *Pyrenophora teres f. teres* (41.95 Mb size, 11,799 genes) [70] and *L. maculans* ‘brassicae’ WA74 (44.20 Mb size, 10,624 genes) [71]. However, the gene number and the genome size of *S. tainanensis* were comparable to *M. oryzae* (on average 12,684 genes, 40.12 Mb size) [72]. In addition, this number is less than the other species in the same genus *Stagonospora*, i.e., model species *S. nodorum* *SN15* (37.02 Mb size, 17,580 genes) (https://www.ncbi.nlm.nih.gov/assembly/GCA_016801405.1/) (accessed on 30 August 2022), substantially higher than the known filamentous fungi though the gene number changed from first 10,792 supported by EST [35] to 12,382 supported by integrated multidimensional omics [73] and now 17,580 [74], while the gene number was comparable to the other typical species in filamentous fungi. These genes encode 12,543 proteins and 97.42% (11,891) of genes were predicted, encoding only one protein isoform. Only 315 genes were predicted with alternatively spliced protein isoforms, including 294 genes encoding two protein isoforms, 20 genes encoding three protein isoforms, and only 1 gene encoding 4 protein isoforms (Table S5). The number of exons per gene ranged from 1~18, and most genes contained 1~5 exons (Table S6). The BUSCO completeness of genes is 99.18% at fungi (*n* = 758) and 99.87% at ascomycota (*n* = 1706) (Figure 4A), indicating high accuracy and completeness of our gene annotations.

The gene distribution is not uniform on the contigs. In most cases, it is opposite to the distribution of repetitive sequences, especially in the repeat-riched telomeric region at both ends of the contig, which contains almost no genes (Figure 3C). This phenomenon is common in repeat-rich pathogenic fungi, such as like rice blast fungus *P. oryzae* [30] and soil borne plant pathogen *Verticillium dahliae* [75].

In addition, no protein-coding gene was identified in the smallest contig ctg12 (154,536 bp). Blastn against with NCBI nr database revealed it is mitochondrion DNA, the most similar sequence is mitochondrion from *B. sorokiniana* (NC_047242.1, 92.83% similarity and 26% coverage). Thus, we did not show it in the genome circos plot (Figure 3C).

### 3.6. General Gene Functional Annotation

W General gene functional annotation estimated that 69.24% (8452) of genes contain conserved protein domains, and 41.95% (5121) were classified by GO terms, with 36.40% (4443) mapping to KEGG pathways and 74.37% (9078) assigned to KOG categories (Table 3 and Tables S7–S10).

The top five GO annotations mainly referred to protein transport and binding activity, including ‘transmembrane transport’ (562 genes), ‘transmembrane transporter activity’ (417 genes), ‘ATP binding’ (466 genes), ‘protein binding’ (446 genes), and ‘zinc ion binding’ (313 genes) (Table S11). The top KEGG annotation referred biosynthesis pathway, including ‘biosynthesis of secondary metabolites’ (325 genes), ‘microbial metabolism in diverse environments’ (179 genes), ‘biosynthesis of cofactors’ (113 genes), and ‘biosynthesis of amino acids’ (105 genes) (Table S12).

**Figure 5:**
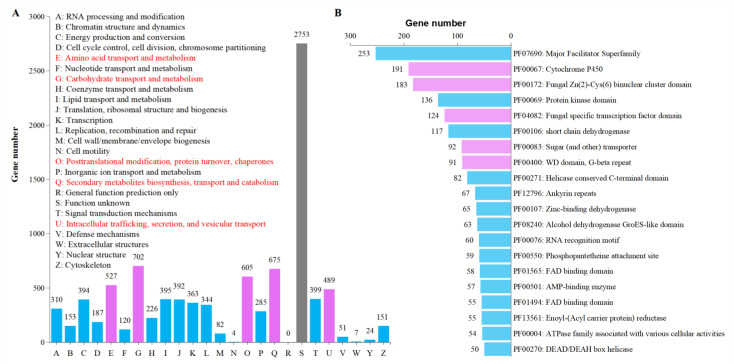
General gene functional annotation. (**A**) KOG annotation and (**B**) Pfam annotation (top20). Red color indicates the top terms associated with candidate pathogenicity-related genes.

KOG annotated genes were divided into 24 categories of which, ‘E: Amino acid transport and metabolism’ (527 genes), ‘G: Carbohydrate transport and metabolism’ (702 genes), ‘O: Posttranslational modification, protein turnover, chaperones’ (605 genes), ‘Q: Secondary metabolites biosynthesis, transport and catabolism’ (675 genes), and ‘U: Intracellular trafficking, secretion, and vesicular transport’ (489 genes) were top five terms associated with candidate pathogenicity-related genes (Figure 5A).

The Pfam annotation revealed a set of candidate pathogenicity-related genes, including ‘Cytochrome P450′ (191 genes) (Table S13), ‘fungal Zn(2)-Cys(6) binuclear cluster domain’ (183 genes), ‘fungal specific transcription factor domain’ (124 genes), ‘Sugar (and other) transporter’ (92 genes), and ‘WD domain, G-beta repeat’ (91 genes) (Figure 5B).

### 3.7. Annotation of Pathogenicity-Related Genes

**Figure 6:**
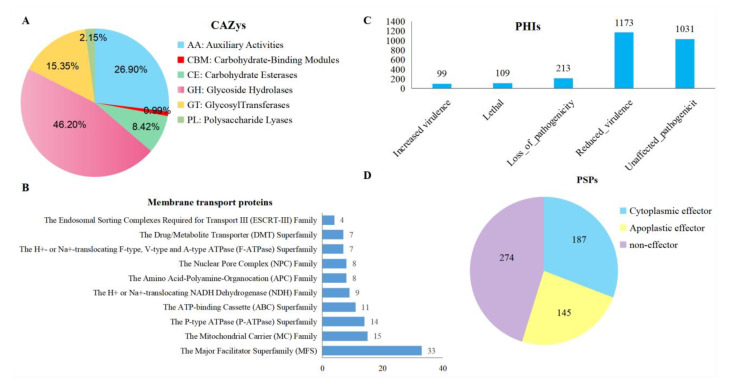
Summary of pathogenicity-related gene annotations. (**A**) CAZys, (**B**) Membrane transport proteins (top10), (**C**) PHIs, and (**D**) Putative secreted proteins.

To understand the pathogenicity mechanism of *S. tainanensis*, we identified a large number of pathogenicity-related proteins, including 599 CAZys, 248 membrane transport proteins, and 2379 PHIs (Table 3), and the number of CAZys was more than in the *M. grisea* (539) [59]. CAZys mainly consisted of 280 (46.20%) glycoside hydrolases (GHs), 163 (26.90%) auxiliary activities (AAs), 93 (15.35%) glycosyl transferases (GTs), and 51 (8.42%) carbohydrate esterases (CEs) (Figure 6A and Table S14). The top three membrane transport proteins included 33 ‘The Major Facilitator Superfamily (MFS)’, 15 ‘The Mitochondrial Carrier (MC) Family’, 14 ‘The P-type ATPase (P-ATPase) Superfamily’, 11 ‘The ATP-binding Cassette (ABC) Superfamily’, and 9 ‘The H^+^ or Na^+^-translocating NADH Dehydrogenase (NDH) Family’ (Figure 6B and Table S15).

Blast analysis of genomic loci using PHI-base revealed a total of 2625 PHI-associated genes, which is much more than the *M. grisea* PMg-ID (868) [74] but less than those in 16 *Sporothrix* strains (from 3083 to 4750) [76]. Among 2625 genes, 99 enhance virulence, 1173 reduce virulence, 109 are lethal, 213 are pathogenicity lost, and 1031 are pathogenicity unaffected (Figure 6C and Table S16).

We identified 1323 proteins with a signal peptide and 609 proteins (encoded by 606 genes) with extracellular location that were defined as putative secreted proteins (PSPs) after removing proteins containing transmembrane helix (Table 3). Among these secreted proteins, we deciphered 333 effectors (encoded by 332 genes), including 187 cytoplasmic and 145 apoplastic effectors (Figure 6D and Table S17), and the number of effectors was much less than that in the *M. grisea* PMg-ID (594) [70]. Interestingly, we found the putative secreted proteins were enriched in or nearby the high repeat regions (Figure 3C), which is consistent with other pathogenic fungi, such as *Magnaporthe oryzae* [46].

### 3.8. Secondary Metabolite Biosynthetic Gene Clusters (SMBGCs)

**Figure 7:**
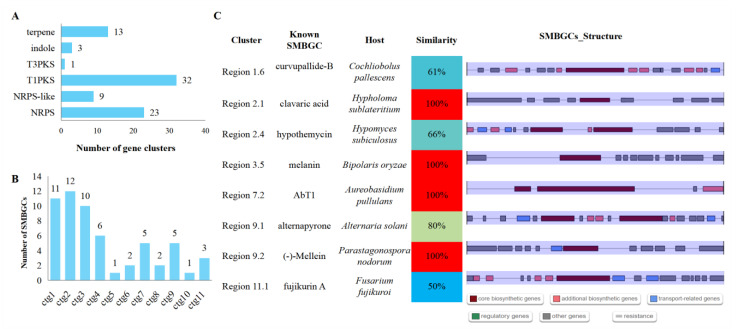
Profiles of secondary metabolite biosynthetic gene clusters. (**A**) Type of SMBGCs. (**B**) Contig distribution of SMBGCs. (**C**) Most similarity with known SMBGCs (similarity ≥ 50%).

A total of 58 SMBGCs were identified, including 23 nonribosomal peptide synthetases (NRPS), 9 NRPS-like, 32 type I polyketide synthases (T1PKS), 1 type III polyketide synthases (T3PKS), 3 indoles, and 13 terpenes (Figure 7A and Table S18). More than half of the SMBGCs (33) were located in ctg1, ctg2, and ctg3, the top three longest contigs (Figure 7B). Eight SMBGCs were found to have more than a 50% similarity with the known SMBGCs, with four having a 100% similarity with the clavaric acid biosynthetic gene cluster from *Hypholoma sublateritium*, melanin biosynthetic gene cluster from *B. oryzae*, AbT1 biosynthetic gene cluster from *Aureobasidium pullulans,* and (-)-Mellein biosynthetic gene cluster from *Parastagonospora nodorum*, respectively (Figure 7C). The rest of the 50 novel SMBGCs will provide a chance for mining novel secondary metabolites in *S. tainanensis*.

Interestingly, clavaric acid was reported to be an inhibitor of the human Ras-farnesyl transferase [77,78]; it thus has antitumor activity [79], and its terpene biosynthetic gene cluster was also detected in *Aspergillus terreus* [80] and *Sporothrix* species [76]. Melanin, a black pigment synthesized by T1PKS type SMBGCs, has a central role in the pathogenicity of plant pathogenic fungi, such as rice blast fungus *P. oryzae* [81,82].

### 3.9. Comparative Genomic Analysis

**Figure 8:**
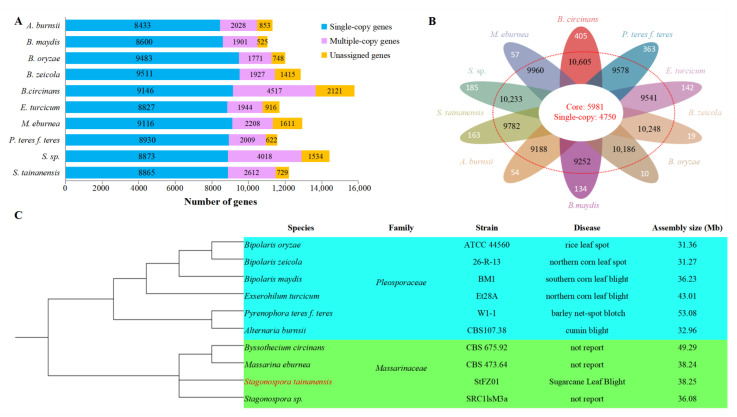
Comparative genomics analysis with close species. (**A**) Genes clustered in orthogroups. (**B**) Summary of orthogroups among species. Species specific genes (white), total orthogroups (black, in the red dotted circle), and core orthogroups (red) were shown from outside to inside of flower. (**C**) Phylogenetic tree inferred with alignment of single-copy core orthogroups.

To figure out the genome differentiation with close relationship species, whole-genome orthologous gene cluster analysis was performed among *S. tainanensis,* three species were from the *Massarinaceae* family and the other six species were from the *Pleosporaceae* family in the *Pleosporales* order (Table S2). We collected 125,793 genes from the 10 species, and 114,719 genes (91.20%) were clustered into 14,038 orthorgroups (Table S19). For the genome of *S. tainanensis* StFZ01, 114,77 out of 12,206 genes (94.03%) were clustered into 9782 orthorgroups, which include 5981 (61.14%) core orthorgroups shared by all other nine fungi, and 47 (0.48%) species-specific orthorgroups with 163 genes (Figure 8A,B and Table S20). Together with the 729 unclustered genes, we identified 892 species-specific genes (7.31%) in *S. tainanensis* StFZ01 (Figure 8A and Table S20).

We selected 4750 single-copy orthorgroups to construct phylogenetic trees (Figure 8C), which showed that *S. tainanensis* and *Stagonospora sp.* were placed on a branch outside of *Massarina eburnea* and *Byssothecium circinans* (Figure 8C).

## 4. Conclusions

In conclusion, this study presented the first T2T chromosome-level genome assembly and high-quality gene annotation of the pathogenic fungus *S. tainanensis* strain StFZ01 causing sugarcane leaf blight, integrating with Nanopore sequencing and Illumina sequencing. The well annotated repeats and genes, such as CAZys and effectors will play as the reference genome for designing species-specific molecular markers and identifying pathogenicity-related genes in the future.

## Figures and Tables

**Table 1:** Genome Assembly Features of *Stagonospora Tainanensis* Strain StFZ01.

Features	StFZ01
Assembly size (bp)	38,252,541
Contig number	12
Contig N50 (bp)	2,858,663
L50	4
Contig N90 (bp)	2,113,312
L90	10
Average contig length (bp)	3,187,712
Maximum contig length (bp)	7,120,155
GC content	51.49%
Repeat sequences	13.20%
Protein-coding genes	12,206
tRNAs	162
rRNAs	142
Other ncRNA	43

**Table 2:** Repeats Identified in *Stagonospora Tainanensis* Strain StFZ01.

	Features	Count	Length (bp)	Percentage (%)
Interspersed repeats	SINEs ^1^	62	12,515	0.03
	LINEs ^2^	288	498,662	1.30
	LTR ^3^ elements	2592	1,927,917	5.04
	DNA transposons	1101	1,242,571	3.25
	Unclassified	3246	882,135	2.31
Tandem repeats	Small RNA	137	111,320	0.29
	Simple repeats	7496	312,397	0.82
	Low complexity	1175	60,609	0.16
	Total repeats	-	5,048,126	13.20

^1^ short interspersed nuclear elements; ^2^ long interspersed nuclear elements; ^3^ long terminal repeat.

**Table 3:** Summary of Gene Functional Annotation.

Annotation	Gene Number	Percentage (%)
Pfam	8452	69.24%
GO ^1^	5121	41.95%
KEGG ^2^	4443	36.40%
KOG ^3^	9078	74.37%
CAZys ^4^	599	4.91%
PHIs ^5^	2379	19.49%
Cytochrome P450 enzymes	191	1.56%
Membrane transport proteins	248	2.03%
Putative secreted proteins	606	4.96%
Effectors	332	2.72%
SMBGCs ^6^	58	0.48%

^1^ Gene Ontology; ^2^ Kyoto Encyclopedia of Genes and Genome; ^3^ EuKaryotic Orthologous Groups; ^4^ Carbohydrate-Active enZymes; ^5^ Pathogen–Host Interaction genes; ^6^ Secondary Metabolite Biosynthetic Gene Clusters.

## Data Availability

The whole genome sequence (GWHBJVM00000000) and the raw sequence data (CRA007499) reported in this paper have been respectively deposited in the WGH (Genome Warehouse, https://ngdc.cncb.ac.cn/gwh) and the GSA (Genome Sequence Archive, https://ngdc.cncb.ac.cn/gsa) in National Genomics Data Center (NGDC), China National Center for Bioinformation (CNCB) [83], under Bioproject PRJCA010492.

## Supplementary Materials

The following supporting information can be downloaded at: https://www.mdpi.com/article/10.3390/jof8101088/s1, Table S1: Summary of sequencing reads; Table S2: Whole-genome proteins date of close species used for orthologous gene clusters; Table S3: Genome size estimated by GenomeScope2 with Illumina genomic reads; Table S4: Contigs with telomeric repeats; Table S5: Distribution of protein isoforms encodes by genes; Table S6: Distribution of exon numbers per gene; Table S7: GO annotation; Table S8: KEGG annotation; Table S9; Pfam Annotation; Table S10: KOG annotation; Table S11: Summary of GO annotation; Table S12: Summary of KEGG pathway annotation; Table S13: Genes with cytochrome P450 annotation; Table S14: CAZys annotation; Table S15: Membrane transport proteins annotated by TCDB; Table S16: PHI_annotation; Table S17: Summary of secreted proteins and effectors; Table S18: Annotation of secondary metabolite biosynthetic gene clusters by AntiSMASH; Table S19: Summary of orthogroups analysis overall species; Table S20: Summary of orthogroups analysis per species.

## References

[1] Li Y.-R., Yang L.-T. (2015). Sugarcane agriculture and sugar industry in China. Sugar Tech.

[2] Xu F., Wang Z.T., Lu G.L., Zeng R.S., Que Y.X. (2021). Sugarcane ratooning ability: Research status, shortcomings, and prospects. Biology.

[3] Raid R.N. (1989). Physiological specialization in sugarcane rust (*Puccinia melanocephala*) in Florida. Plant Dis..

[4] Rajput M.A., Rajput N.A., Syed R.N., Lodhi A.M., Que Y. (2021). Sugarcane smut: Current knowledge and the way forward for management. J. Fungi.

[5] Hoy J.W., Hollier C.A. (2009). Effect of brown rust on yield of sugarcane in Louisiana. Plant Dis..

[6] Shan H., Li W., Zhang R., Wang X., Li J., Cang X., Yin J., Luo Z., Huang Y. (2018). Analysis on epidemic reason of sugarcane pokahh boeng and its losses on yield and sucrose content. Sugar Crop China.

[7] Patil A.S., Hapase D.G. (1987). Studies on pokkah boeng disease of sugarcane in Maharashtra. Indian Phytopathol..

[8] Vishwakarma S.K., Kumar P., Nigam A., Singh A., Kumar A. (2013). Pokkah boeng: An emerging disease of sugarcane. J. Plant Pathol. Microb..

[9] Viswanathan R. (2018). Changing scenario of sugarcane diseases in India since introduction of hybrid cane varieties: Path travelled for a century. J. Sugarcane Res..

[10] Comstock J.C., Glynn N.C., Davidson R.W. (2010). Sugarcane rusts in Florida. Proc. Inter. Soc. Sugar Cane Tech..

[11] Selvakumar R., Viswanathan R., Viswanathan R., Sundar R.A., Malathi P., Selvakumar R., Jayakumar V., Nithya K. (2018). History of sugarcane rusts in India. Res Accomplishments in Sugarcane Pathology.

[12] Comstock J.C., Sood S.G., Glynn N.C., Shine J.M., McKemy J.M., Castlebury L.A. (2008). First report of *Puccinia kuehnii*, causal agent of orange rust of sugarcane, in the United States and western hemisphere. Plant Dis..

[13] Viswanathana R., Ashwin N.M.R. (2020). Brown spot of sugarcane: An emerging disease in South Western region in India. J. Sugarcane Res..

[14] Patel R.R., Patel D.D., Bhatt J., Thakor P., Triplett L.R., Thakkar V.R. (2021). Induction of pre-chorismate, jasmonate and salicylate pathways by Burkholderia sp. RR18 in peanut seedlings. J. Appl. Microbiol..

[15] Patel R.R., Thakkar V.R., Subramanian B.R. (2015). A *Pseudomonas guariconensis* strain capable of promoting growth and controlling collar rot disease in *Arachis hypogaea* L.. Plant Soil.

[16] Wang X.Y., Li J., Yang K., Shan H.L., Zhang R.Y., Wang C.M., Cang X.Y., Yin J., Luo Z.M., Li W.F. (2021). Evaluation of resistance to brown streak disease in new and main cultivated sugarcane varieties. Acta Phytopathol. Sin..

[17] Yen W.Y., Chi C.C. (1952). Studies on leaf blight of sugarcane (I). J. Sugarcane Res. Taiwan.

[18] Hsieh W.H. (1997). The Causal organism of sugarcane leaf blight. Mycologia.

[19] Shah D.A., Bergstrom G.C. (2002). A rainfall-based model for predicting the regional incidence of wheat seed infection by *Stagonospora nodorum* in New York. Phytopathology.

[20] Abeysekara N.S., Friesen T.L., Keller B., Faris J.D. (2009). Identification and characterization of a novel host-toxin interaction in the wheat-Stagonospora nodorum pathosystem. Theor. Appl. Genet..

[21] Solomon P.S., Lowe R.G.H., Tan K.-C., Waters O.D.C., Oliver R.P. (2006). *Stagonospora nodorum*: Cause of *stagonospora nodorum* blotch of wheat. Mol. Plant Pathol..

[22] Hsieh W.H., Comstock J.C., Rott P., Bailey R.A., Comstock J.C., Croft A., Saumtally A.S. (2000). Leaf Blight. A Guide to Sugarcane Diseases.

[23] O’Neill N.R., Farr D.F. (1996). Miscanthus blight, a new foliar disease of ornamental grasses and sugarcane incited by *Leptosphaeria sp*. and its anamorphic state *Stagonospora sp.*. Plant Dis..

[24] Ren H. (2022). Isolation, Genome Assembly and Molecular Detection Method of the Pathogen Causing Sugarcane Leaf Blight. Master’s Thesis.

[25] Wang Z., Ren H., Pang C., Lu G., Xu F., Cheng W., Que Y., Xu L. (2022). An autopolyploid-suitable polyBSA-seq strategy for screening candidate genetic markers linked to leaf blight resistance in sugarcane. Theor. Appl. Genet..

[26] Wang Z., Lu G., Wu Q., Li A., Que Y., Xu L. (2022). Isolating QTL controlling sugarcane leaf blight resistance using a two-way pseudo-testcross strategy. Crop J..

[27] Tedersoo L., Albertsen M., Anslan S., Callahan B. (2021). Perspectives and benefits of high-throughput long-read sequencing in microbial ecology. Appl. Environ. Microbiol..

[28] Aragona M., Haegi A., Valente M.T., Riccioni L., Orzali L., Vitale S., Luongo L., Infantino A. (2022). New-generation sequencing technology in diagnosis of fungal plant pathogens: A dream comes true?. J. Fungi.

[29] Guiglielmoni N., Houtain A., Derzelle A., Van Doninck K., Flot J.F. (2021). Overcoming uncollapsed haplotypes in long-read assemblies of non-model organisms. BMC Bioinform..

[30] Bao J., Chen M., Zhong Z., Tang W., Lin L., Zhang X., Jiang H., Zhang D., Miao C., Tang H. (2017). PacBio sequencing reveals transposable elements as a key contributor to genomic plasticity and virulence variation in *Magnaporthe oryzae*. Mol. Plant.

[31] Zhong Z., Chen M., Lin L., Han Y., Bao J., Tang W., Lin L., Lin Y., Somai R., Lu L. (2018). Population genomic analysis of the rice blast fungus reveals specific events associated with expansion of three main clades. ISME J..

[32] Kelly A.C., Ward T.J. (2018). Population genomics of *Fusarium graminearum* reveals signatures of divergent evolution within a major cereal pathogen. PLoS ONE.

[33] Alouane T., Rimbert H., Bormann J., Gonzalez-Montiel G.A., Loesgen S., Schafer W., Freitag M., Langin T., Bonhomme L. (2021). Comparative genomics of eight *Fusarium graminearum* strains with contrasting aggressiveness reveals an expanded open pangenome and extended effector content signatures. Int. J. Mol. Sci..

[34] Feng Z., Hsiang T., Liang X., Zhang R., Sun G. (2021). Draft genome sequence of cumin blight pathogen *Alternaria burnsii*. Plant Dis..

[35] Haridas S., Albert R., Binder M., Bloem J., LaButti K., Salamov A., Andreopoulos B., Baker S.E., Barry K., Bills G. (2020). 101 Dothideomycetes genomes: A test case for predicting lifestyles and emergence of pathogens. Stud. Mycol..

[36] Zeiner C.A., Purvine S.O., Zink E.M., Pasa-Tolic L., Chaput D.L., Haridas S., Wu S., LaButti K., Grigoriev I.V., Henrissat B. (2016). Comparative analysis of secretome profiles of manganese (II)-oxidizing ascomycete fungi. PLoS ONE.

[37] Hane J.K., Lowe R.G., Solomon P.S., Tan K.C., Schoch C.L., Spatafora J.W., Crous P.W., Kodira C., Birren B.W., Galagan J.E. (2007). Dothideomycete plant interactions illuminated by genome sequencing and EST analysis of the wheat pathogen *Stagonospora nodorum*. Plant Cell.

[38] Ranallo-Benavidez T.R., Jaron K.S., Schatz M.C. (2020). GenomeScope 2.0 and Smudgeplot for reference-free profiling of polyploid genomes. Nat. Commun..

[39] Kokot M., Dlugosz M., Deorowicz S. (2017). KMC 3: Counting and manipulating k-mer statistics. Bioinformatics.

[40] Hu J., Fan J., Sun Z., Liu S. (2020). NextPolish: A fast and efficient genome polishing tool for long-read assembly. Bioinformatics.

[41] Manni M., Berkeley M.R., Seppey M., Simao F.A., Zdobnov E.M. (2021). BUSCO Update: Novel and streamlined workflows along with broader and deeper phylogenetic coverage for scoring of eukaryotic, prokaryotic, and viral genomes. Mol. Biol. Evol..

[42] Kim D., Paggi J.M., Park C., Bennett C., Salzberg S.L. (2019). Graph-based genome alignment and genotyping with HISAT2 and HISAT-genotype. Nat. Biotechnol..

[43] Li H. (2018). Minimap2: Pairwise alignment for nucleotide sequences. Bioinformatics.

[44] Jung Y., Han D. (2022). BWA-MEME: BWA-MEM emulated with a machine learning approach. Bioinformatics.

[45] Nurk S., Koren S., Rhie A., Rautiainen M., Bzikadze A.V., Mikheenko A., Vollger M.R., Altemose N., Uralsky L., Gershman A. (2022). The complete sequence of a human genome. Science.

[46] Flynn J.M., Hubley R., Goubert C., Rosen J., Clark A.G., Feschotte C., Smit A.F. (2020). RepeatModeler2 for automated genomic discovery of transposable element families. Proc. Natl. Acad. Sci. USA.

[47] Bruna T., Hoff K.J., Lomsadze A., Stanke M., Borodovsky M. (2021). BRAKER2: Automatic eukaryotic genome annotation with GeneMark-EP+ and AUGUSTUS supported by a protein database. NAR Genom. Bioinform..

[48] Hoff K.J., Stanke M. (2019). Predicting genes in single genomes with AUGUSTUS. Curr. Protoc. Bioinform..

[49] Bruna T., Lomsadze A., Borodovsky M. (2020). GeneMark-EP+: Eukaryotic gene prediction with self-training in the space of genes and proteins. NAR Genom. Bioinform..

[50] Chan P.P., Lin B.Y., Mak A.J., Lowe T.M. (2021). tRNAscan-SE 2.0: Improved detection and functional classification of transfer RNA genes. Nucleic Acids Res..

[51] Nawrocki E.P., Eddy S.R. (2013). Infernal 1.1: 100-fold faster RNA homology searches. Bioinformatics.

[52] Kalvari I., Nawrocki E.P., Ontiveros-Palacios N., Argasinska J., Lamkiewicz K., Marz M., Griffiths-Jones S., Toffano-Nioche C., Gautheret D., Weinberg Z. (2021). Rfam 14: Expanded coverage of metagenomic, viral and microRNA families. Nucleic Acids Res..

[53] Jones P., Binns D., Chang H.Y., Fraser M., Li W., McAnulla C., McWilliam H., Maslen J., Mitchell A., Nuka G. (2014). InterProScan 5: Genome-scale protein function classification. Bioinformatics.

[54] Cantalapiedra C.P., Hernandez-Plaza A., Letunic I., Bork P., Huerta-Cepas J. (2021). eggNOG-mapper v2: Functional Annotation, Orthology Assignments, and Domain Prediction at the Metagenomic Scale. Mol. Biol. Evol..

[55] Aramaki T., Blanc-Mathieu R., Endo H., Ohkubo K., Kanehisa M., Goto S., Ogata H. (2020). KofamKOALA: KEGG Ortholog assignment based on profile HMM and adaptive score threshold. Bioinformatics.

[56] Buchfink B., Reuter K., Drost H.G. (2021). Sensitive protein alignments at tree-of-life scale using DIAMOND. Nat. Methods.

[57] Mistry J., Finn R.D., Eddy S.R., Bateman A., Punta M. (2013). Challenges in homology search: HMMER3 and convergent evolution of coiled-coil regions. Nucleic Acids Res..

[58] Almagro Armenteros J.J., Tsirigos K.D., Sonderby C.K., Petersen T.N., Winther O., Brunak S., von Heijne G., Nielsen H. (2019). SignalP 5.0 improves signal peptide predictions using deep neural networks. Nat. Biotechnol..

[59] Moller S., Croning M.D., Apweiler R. (2001). Evaluation of methods for the prediction of membrane spanning regions. Bioinformatics.

[60] Sperschneider J., Dodds P.N. (2022). EffectorP 3.0: Prediction of apoplastic and cytoplasmic effectors in fungi and Oomycetes. Mol. Plant Microbe Interact..

[61] Blin K., Shaw S., Kloosterman A.M., Charlop-Powers Z., van Wezel G.P., Medema M.H., Weber T. (2021). antiSMASH 6.0: Improving cluster detection and comparison capabilities. Nucleic Acids Res..

[62] Emms D.M., Kelly S. (2019). OrthoFinder: Phylogenetic orthology inference for comparative genomics. Genome Biol..

[63] Katoh K., Standley D.M. (2013). MAFFT multiple sequence alignment software version 7: Improvements in performance and usability. Mol. Biol. Evol..

[64] Price M.N., Dehal P.S., Arkin A.P. (2010). FastTree 2--approximately maximum-likelihood trees for large alignments. PLoS ONE.

[65] Letunic I., Bork P. (2021). Interactive tree of life (iTOL) v5: An online tool for phylogenetic tree display and annotation. Nucleic Acids Res..

[66] Wang Y., Kang H., Yao J., Li Z., Xia X., Zhou S. (2022). An improved genome sequence resource of *Bipolaris maydis*, causal agent of Southern corn leaf blight. Phytopathology.

[67] O’Connell R.J., Thon M.R., Hacquard S., Amyotte S.G., Kleemann J., Torres M.F., Damm U., Buiate E.A., Epstein L., Alkan N. (2012). Lifestyle transitions in plant pathogenic *Colletotrichum* fungi deciphered by genome and transcriptome analyses. Nat. Genet..

[68] Syauqi J., Chen R.K., Cheng A.H., Wu Y.F., Chung C.L., Lin C.C., Chou H.P., Wu H.Y., Jian J.Y., Liao C.T. (2022). Surveillance of rice blast resistance effectiveness and emerging virulent isolates in Taiwan. Plant Dis..

[69] Reddy B., Mehta S., Prakash G., Sheoran N., Kumar A. (2022). Structured framework and genome analysis of *Magnaporthe grisea* inciting pearl millet blast disease reveals versatile metabolic pathways, protein families, and virulence factors. J. Fungi.

[70] Ellwood S.R., Liu Z., Syme R.A., Lai Z., Hane J.K., Keiper F., Moffat C.S., Oliver R.P., Friesen T.L. (2010). A first genome assembly of the barley fungal pathogen *Pyrenophora Teres* F. *Teres*. Genome Biol..

[71] Grandaubert J., Lowe R.G.T., Soyer J.L., Schoch C.L., Van de Wouw A.P., Fudal I., Robbertse B., Lapalu N., Links M.G., Ollivier B. (2014). Transposable element-assisted evolution and adaptation to host plant within the *Leptosphaeria maculans*-*Leptosphaeria biglobosa* species complex of fungal pathogens. BMC Genom..

[72] Devanna B.N., Jain P., Solanke A.U., Das A., Thakur S., Singh P.K., Kumari M., Dubey H., Jaswal R., Pawar D. (2022). Understanding the dynamics of blast resistance in rice-*Magnaporthe oryzae* interactions. J. Fungi.

[73] Hane J.K., Williams A., Oliver R.P., Poggeler S., Wostemeyer J. (2011). Genomic and comparative analysis of the class Dothideomycetes. The Mycota.

[74] Bertazzoni S., Jones D.A.B., Phan H.T., Tan K.C., Hane J.K. (2021). Chromosome-level genome assembly and manually-curated proteome of model necrotroph *Parastagonospora nodorum* Sn15 reveals a genome-wide trove of candidate effector homologs, and redundancy of virulence-related functions within an accessory chromosome. BMC Genom..

[75] Faino L., Seidl M.F., Shi-Kunne X., Pauper M., van den Berg G.C., Wittenberg A.H., Thomma B.P. (2016). Transposons passively and actively contribute to evolution of the two-speed genome of a fungal pathogen. Genome Res..

[76] Huang M., Ma Z., Zhou X. (2020). Comparative genomic data provide new insight on the evolution of pathogenicity in *Sporothrix* species. Front. Microbiol..

[77] Jayasuriya H., Silverman K.C., Zink D.L., Jenkins R.G., Sanchez M., Pelaez F., Vilella D., Lingham R.B., Singh S.B. (1998). Clavaric acid: A triterpenoid inhibitor of farnesyl-protein transferase from *Clavariadelphus truncatus*. J. Nat. Prod..

[78] Lingham R.B., Silverman K.C., Jayasuriya H., Kim B.M., Amo S.E., Wilson F.R., Rew D.J., Schaber M.D., Bergstrom J.D., Koblan K.S. (1998). Clavaric acid and steroidal analogues as Ras-and FPP-directed inhibitors of human farnesyl-protein transferase. J. Med. Chem..

[79] Godio R.P., Fouces R., Martin J.F. (2007). A squalene epoxidase is involved in biosynthesis of both the antitumor compound clavaric acid and sterols in the basidiomycete *H. sublateritium*. Chem. Biol..

[80] Du X., Li H., Qi J., Chen C., Lu Y., Wang Y. (2021). Genome mining of secondary metabolites from a marine-derived *Aspergillus terreus* B12. Arch. Microbiol..

[81] Zhu S., Yan Y., Qu Y., Wang J., Feng X., Liu X., Lin F., Lu J. (2021). Role refinement of melanin synthesis genes by gene knockout reveals their functional diversity in *Pyricularia oryzae* strains. Microbiol. Res..

[82] Freitas D.F., Rocha I.M., Vieira-da-Motta O., de Paula Santos C. (2021). The role of melanin in the biology and ecology of nematophagous Fungi. J. Chem. Ecol..

[83] Members C.-N., Partners (2021). Database resources of the National Genomics Data Center, China National Center for Bioinformation in 2021. Nucleic Acids Res..

